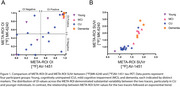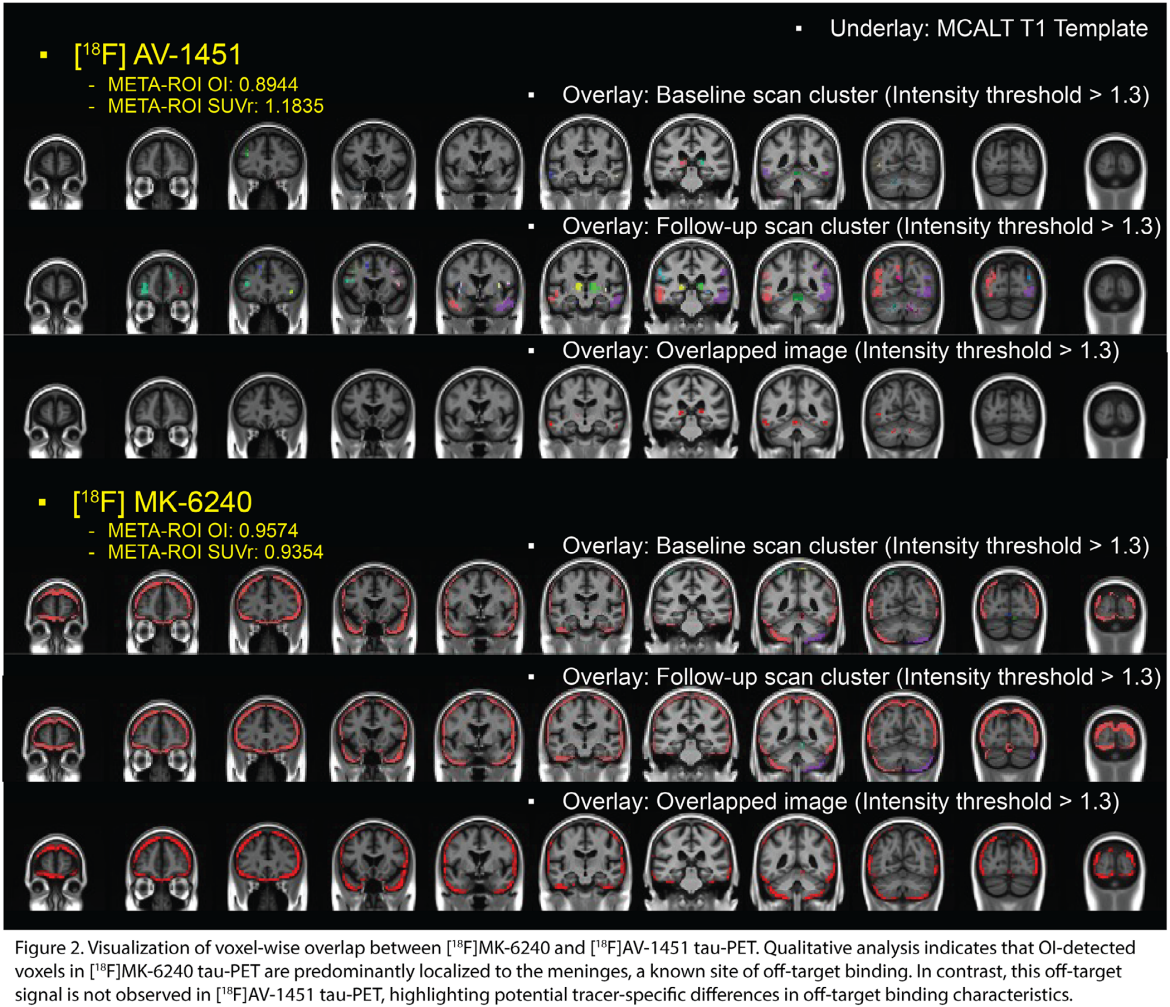# Evaluation discrepancy between [^18^F]MK‐6240 and [^18^F]AV‐1451 tau‐PET using tau‐PET overlap index

**DOI:** 10.1002/alz70856_106145

**Published:** 2026-01-11

**Authors:** Seokbeen Lim, Hoon‐Ki Min, Jessica L. Brunn, David N. Soleimani‐Meigooni, Hwamee Oh, Juan Fortea, Belen Pascual, Brian A. Gordon, Pedro Rosa‐Neto, Suzanne L. Baker, Firoza Z Lussier, Guilherme Povala, Tharick A Pascoal, Val J Lowe

**Affiliations:** ^1^ Mayo Clinic, Rochester, MN, USA; ^2^ Department of Radiology, Mayo Clinic, Rochester, MN, USA; ^3^ University of California, San Francisco, San Francisco, CA, USA; ^4^ Brown University, Providence, RI, USA; ^5^ Hospital de la Santa Creu i Sant Pau, Barcelona, Barcelona, Spain; ^6^ Houston Methodist Research Institute, Houston, TX, USA; ^7^ Washington University in St. Louis, St. Louis, MO, USA; ^8^ Translational Neuroimaging Laboratory, The McGill University Research Centre for Studies in Aging, Montréal, QC, Canada; ^9^ Lawrence Berkeley National Laboratory, Berkeley, CA, USA; ^10^ University of Pittsburgh, Pittsburgh, PA, USA

## Abstract

**Background:**

The overlap index (OI), previously introduced by Lee et al. (2022), has proven to be a reliable method for detecting tau accumulation via tau‐PET imaging with flortaucipir ([^18^F]AV‐1451). This technique identifies voxel‐wise increases in standardized uptake value ratio (SUVr) across serial scans. However, the relationship between tau‐PET measurements obtained with [^18^F]MK‐6240 and [^18^F]AV‐1451 using the OI remains unclear. This study aims to investigate the relationship between [^18^F]MK‐6240 and [^18^F]AV‐1451 tau‐PET using the tau‐PET OI.

**Method:**

The study included 27 participants from the HEAD project, all of whom underwent two serial tau‐PET scans (each [^18^F]MK‐6240 and [^18^F]AV‐1451) along with 3T T1‐weighted MRI, acquired within an average interval of 18 months. SUVr maps for each tau‐PET tracer were normalized to the cerebellar crus grey matter, and MR images were co‐registered to the MCALT T1 template. Tau‐PET images were spatially resampled to the template space, and OI was computed within a predefined META‐ROI using an intensity threshold of 1.3 and a cutoff of 0.5. Clusters containing fewer than 20 contiguous voxels were excluded from analysis. The META‐ROI SUVr was calculated as the average SUVr across the selected region's SUVr. Additionally, a visual comparison was performed to assess the spatial overlap of OI‐identified voxels between the two tau‐PET tracers.

**Result:**

OI values within the META‐ROI exhibited a broad distribution between [^18^F]MK‐6240 and [^18^F]AV‐1451 tau‐PET, particularly in cognitively unimpaired (CU) and younger individuals (Figure 1A). Unlike the OI distribution, the relationship between META‐ROI SUVr values of both tracers followed an exponential trend (Figure 1B). Visual inspection revealed that OI‐detected voxels in [^18^F]MK‐6240 tau‐PET were frequently localized to the meninges, an established site of off‐target binding, which was not observed in [^18^F]AV‐1451 tau‐PET (Figure 2).

**Conclusion:**

The association between [^18^F]MK‐6240 and [^18^F]AV‐1451 tau‐PET, as assessed using the OI, exhibited considerable variability in CU and younger participants. Additionally, the presence of overlapping voxels in the meninges in [^18^F]MK‐6240 tau‐PET highlights the need to account for and potentially exclude off‐target binding effects in these populations. The influence of meningeal signal and other off‐target binding on the OI in the temporal META‐ROI needs to be further evaluated.